# Phantom Force Balance Procedure for Predicting the Modulus of Entangled Polymer Networks

**DOI:** 10.1021/acspolymersau.5c00036

**Published:** 2025-08-05

**Authors:** Tim Bernhard, Andrei A. Gusev

**Affiliations:** † Laboratory for Nanometallurgy, Department of Materials, 111950 ETH Zürich, 8093 Zürich, Switzerland; ‡ Department of Materials, 111950 ETH Zürich, 8093 Zürich, Switzerland

**Keywords:** end-linked polymer networks, entanglements, Monte Carlo network generation, force balance homogenization procedure, equilibrium shear modulus

## Abstract

A computational phantom Force Balance, Maximum Entropy Homogenization Procedure is presented to predict the equilibrium shear modulus of entangled polymer networks. A Monte Carlo method is used to generate periodic bead–spring microstructures of polymer networks. Entanglements are introduced by merging two internal beads of adjacent network strands, yielding additional tetrafunctional cross-links. The microstructures are optimized to their minimum free energy state, for which the modulus is readily available. The procedure is validated by comparing its modulus predictions with those from both stress–relaxation molecular dynamics (MD) simulations and the Miller–Macosko theory (MMT). Near-perfect agreement with both the MD and MMT results is obtained, with the required computational resources being about four and more orders of magnitude smaller than those in the MD simulations. Finally, good agreement with experimental results over a variety of different polymer networks is demonstrated, including those with bottlebrush and comb-like polymer strands and also near-critical gels, suggesting that the presented procedure can be practically used to predict the modulus of arbitrary polymer networks.

## Graphical abstract

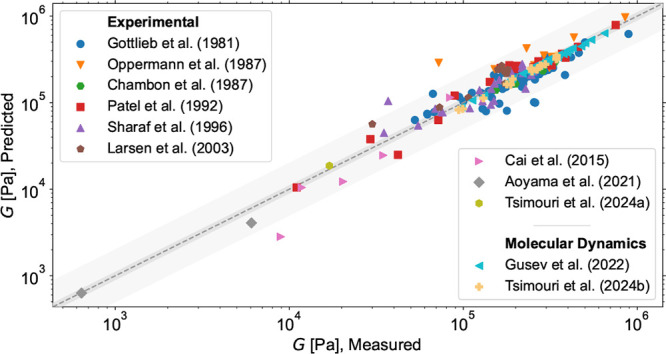

## 1 Introduction

Polymers are ubiquitous in everyday life, appearing in both natural and synthetic forms. Natural polymers, such as keratin in wool and cellulose in cotton, are widely used in textiles,^1^ while synthetic polymers such as PET serve various purposes, including clothing, bottles, and other containers.^2^ Over time, polymer applications have expanded significantly, encompassing fields such as soft robotics,^3^ stretchable electronics,^4^ and stimuli-responsive gels.^5,6^ The unique elastic and viscoelastic properties^7,8^ exhibited by polymer networks and gels have made them essential in applications such as vibration and acoustic damping.^9–24^ Advances in polymer science have also led to innovations in biological and medical fields, with polymers being employed in tissue engineering scaffolds,^25^ drug delivery systems,^26,27^ and bioinspired materials.^28^

Recently, interest has increased in near-critical polymer networks, which refer to networks with a degree of polymerization *p* just past the gel point, the point at which the fluidity of the polymer melt is lost during the cross-linking process.^29^ These very soft polymer networks have many applications, such as touch sensors,^30^ adhesives, drug delivery, cell scaffolds, and solid electrolytes.^31^ Current interest is also driven by the ability of soft polymer networks to mimic the behavior of biological tissue.^32^ Specifically, reproducing the stress–strain characteristics of muscle and skin fibers using polymers can greatly enhance their applications in medicine and health technology,^32,33^ including cell and tissue organization and organ regeneration,^34–36^ as waveguides for photomedicine,^37,38^ or for controlled release of therapeutic agents in defined doses over long periods of time.^39,40^

The equilibrium shear modulus *G*_eq_ of polymer networks and gels is on the order of 10^2^–10^6^ Pa, whereas their bulk modulus is about 10^9^ Pa, which is similar to that of liquid water under ambient conditions.^32,41,42^ Therefore, to describe the viscoelastic properties of isotropic polymer networks, gels and melts, one commonly uses an incompressible approximation with a single shear relaxation modulus *G*(*t*),^11,29,43,44^ which is defined from the relaxation of shear stress τ­(*t*) after a small step shear strain γ imposed at time *t* = 0 as *G*(*t*) = τ­(*t*)/γ. For *t* → *∞*, this becomes the equilibrium shear modulus *G*_eq_, also sometimes referred to as the plateau modulus. For brevity, from this point forward we will use for *G*_eq_ simply the modulus *G*.

The elasticity of polymer networks has an entropic origin, which was first described using theoretical models with fluctuating phantom Gaussian strands, where phantom refers to the fact that the strands can freely cross each other and themselves. In the 1940s, James and Guth^45–47^ derived a general theoretical formula for the modulus of phantom Gaussian polymer networks, given by

$$G_{\mathrm{ANT}}=\Gamma \nu k_{B}T\;\mathrm{with}\;\Gamma =\langle \gamma_{\alpha }\rangle =\left\langle \frac{{\overset{®}{R}}_{\alpha }^{2}}{R_{\alpha }^{2}}\right\rangle$$ 1

where ν is the number-density of the network strands, *k*_B_ and *T* are the Boltzmann constant and the temperature, respectively, γ_α_ is the chain-dimension factor of strand α, **R̅**_α_ is the mean end-to-end vector, *R*_α_^2^ is the mean-square end-to-end distance of strand α in the ideal state (in a melt), the angular brackets denote the ensemble average over the strands, and the ANT stands for the Affine Network Theory.^42,45–47^

The ANT formula is valid for any phantom Gaussian polymer network and accounts for any possible topological defects. For example, for the strands in branched dangling and soluble molecular structures and primary loops, the mean end-to-end vectors **R̅**_α_ = 0, so γ_α_ = 0, and, as expected, these strands do not contribute to the modulus. However, in general, a detailed molecular model of the polymer network is required to obtain the chain dimension factors γ_α_ of the strands. Such 3D computer models are now routinely available (for a recent review, see Sen and Olsen^48^), and they can be used to calculate the topological factor Γ by considering the ground state of the computer models where the forces exerted on the cross-links by the connected strands are balanced to zero.^42^ The ANT formula [Disp-formula eq1] was computationally validated by comparing its predictions with the results of stress-relaxation molecular dynamics (MD) simulations, using one and the same periodic end-linked Monte Carlo (MC) networks with 10^5^ phantom bead–spring strands.^49^ Note that the ANT formula [Disp-formula eq1] explicitly assumes that the network strands can sample all their available conformations, so it cannot be used for glassy polymer networks, where conformational chain motions are suppressed by the excluded volume interactions.

Using simplified model topological arrangements, different theoretical models have been developed for the modulus of phantom polymer networks, including the affine network model (ANM), the phantom network model (PNM), the Miller-Macosko theory (MMT), and the real elastic network theory (RENT), which are briefly reviewed below.

In the affine network model (ANM),^50^ the vectors **R̅**_α_ of the strands are assumed to follow the Gaussian distribution with the same mean-square end-to-end distances *R*_α_^2^ as in a melt. The mean cross-links positions are assumed to displace affinely with the macroscopic deformation, see eq 10, as they were attached to some elastic background.^29,51–53^ Under such assumptions, the topological factors of the strands are γ_α_ = 1 so Γ = 1 and hence^29,50,54,55^

$$G_{\mathrm{ANM}}=\nu k_{B}T$$ 2

In the phantom network model (PNM),^29,46,54,56^ the cross-links can fluctuate around their mean positions, which reduces the effective stretching of the strands and thus lowers the modulus of the network. The PNM modulus is given by

$$G_{\mathrm{PNM}}=\left(1-\frac{2}{f}\right)\nu k_{B}T$$ 3

where *f* is the functionality of the cross-links. The PNM has been shown to provide accurate estimates of the modulus of well-developed end-linked polymer networks formed from bulk.^42,49^

The ANM and PNM can be viewed as simple one-chain models of ideal tree-like polymer networks without any topological defects such as dangling and soluble structures and loops. And these two models can also be considered as approximations of the ANT formula [Disp-formula eq1], each with its own topological factor Γ = 1 and Γ = 1 – 1/*f*, respectively.

The PNM has traditionally served as a starting point for developing theoretical models that account for the topological defects of real polymer networks and gels. As a classical example, based on Flory’s ideal network assumptions, the MMT accounts for the effect of branched dangling and soluble molecular structures on the modulus of tree-like end-linked polymer networks without loops.^57^ As a recent important contribution, the RENT quantifies the effect of loops on the modulus of phantom polymer networks and gels.^53,58–64^

In real networks the strands cannot pass through one another and hence the entanglements caused by topological restrictions of neighboring chains should be considered. Following on from the works of Busse,^65^ Treloar,^66^ Langley,^67^ Dossin and Graessley,^68^ and Edwards et al.,^69–71^ it is now commonly assumed that the entanglement modulus *G*_e_ and the phantom modulus *G*_ph_ are additive:^49,51,52,72–79^

$$G=G_{e}+G_{\mathrm{ph}}=p_{\mathrm{el}}^{2}G_{e}\left(1\right)+\Gamma \nu k_{B}T$$ 4

where *G*_e_(1) is the plateau modulus of a melt of high molar mass linear polymer chains, *p*_el_ is the probability that a randomly chosen network strand is elastically effective, and it is assumed that the topological factors Γ of real and their topologically equivalent phantom networks are identical.^49^ It has been shown using stress-relaxation MD simulations that the additivity assumption holds well for both vulcanized and end-linked polymer networks formed from bulk.^49,79^

The obvious advantage of these theories is that they provide closed-form solutions for the modulus. However, their extension to complex architecture entangled polymer networks, such as, for example, networks with entangled bottlebrush or comb-like strands,^24,32,80^ does not look straightforward and may require considering large topological arrangements.

Here, we take an alternative route and incorporate the entanglements, treated as additional tetrafunctional cross-links, directly into the phantom ANT description. We use a Force Balance, Maximum Entropy Homogenization Procedure (MEHP) to obtain the ground state positions of both cross-links and entanglements and use them to predict the chain dimension factors γ_α_ of the strands and then the modulus using the ANT formula [Disp-formula eq16]. The procedure is validated by comparing the predicted moduli with those obtained by stress-relaxation MD simulations,^49,81^ using one and the same periodic MD microstructures of entangled end-linked polydimethylsiloxane (PDMS) networks with 10^4^ strands. We show that for fast and accurate predictions, one can alternatively use the MC microstructures.^42^ And we use such representative MC microstructures to compare our computational Force Balance predictions with experimental literature data on the modulus of a variety of real PDMS networks.^24,49,80,82–88^

## 2 Theory for the Modulus of Entangled Polymer Networks

Consider an arbitrary Gaussian polymer network consisting of phantom entropic strands connecting cross-links, which may be either actual chemical cross-links or entanglements, with the latter modeled as tetrafunctional cross-links, and let **R**_*ab*_ = **R**_*b*_ – **R**_*a*_ be the end-to-end vector of a strand connecting two cross-links fixed at positions **R**_*a*_ and **R**_*b*_. The mean force **f**_*ab*_ exerted by this strand on cross-link *a* is given by^7,8,29,43^

$$f_{ab}=\frac{3k_{B}T}{R_{ab}^{2}}R_{ab}$$ 5

where *R*_*ab*_^2^ is the mean-square end-to-end distance of strand *ab* in the ideal state (in a melt). However, given that two cross-links may in general be connected by more than one strand (e.g., by two strands in a secondary loop), it is more consistent to label the network strands using their own number α and write the above force–displacement relation as

$$f_{\alpha }=\frac{3k_{B}T}{R_{\alpha }^{2}}R_{\alpha }$$ 6

In equilibrium the cross-links fluctuate around their mean positions **R̅**_*a*_, and it was proven by James and Guth^45^ that for the Gaussian networks the mean positions **R̅**_*a*_ can be obtained by minimizing the potential energy of an equivalent, classical elastic-thread network with the potential energy

$$U\left(R_{1},\cdot \cdot \cdot ,R_{K}\right)=\frac{1}{2}\underset{\alpha }{\sum }\frac{3k_{B}T}{R_{\alpha }^{2}}R_{\alpha }^{2}$$ 7

where *K* is the total number of cross-links and the summation is carried out over all strands of the network. In the minimum-energy state, the forces acting on the cross-links are balanced to zero^42,45^ :

$$f_{a}=-\frac{\partial U\left(R_{1},\cdot \cdot \cdot ,R_{K}\right)}{\partial R_{a}}=0$$ 8

and the resulting cross-link positions **R̅**_*a*_ determine the topological factor Γ, and thus the network modulus, through the ANT formula [Disp-formula eq16].

The condition that *U* shall be a minimum is equivalent to the condition that the number of configurations (microstates) Ω available for a phantom Gaussian network with fixed cross-links shall be a maximum.^42,45^ Therefore, the positions **R̅**_*a*_ obtained via the Force Balance procedure maximize the network entropy *S* = *k*_B_ log Ω, and thus define the observable equilibrium thermodynamic properties of the network, including its shear modulus.^42,45^ This equivalence was used in our previous works to develop and validate a Force Balance, Maximum Entropy Homogenization Procedure for predicting the shear modulus of unentangled phantom Gaussian polymer networks.^42,89^ A key novelty of the present work is the extension of this approach to entangled polymer networks, in which entanglements are modeled as additional tetrafunctional cross-links.

The stress tensor **σ** of a macroscopically isotropic elastic-thread network consists of two parts: a stress term due to strand stretching and a pressure term arising from excluded volume interactions, which are not explicitly considered in the phantom network description:

$$\sigma_{ij}=\frac{1}{V}\sum_{\alpha }f_{\alpha i}{\overset{®}{R}}_{\alpha j}-P\delta_{ij}=\frac{3k_{B}T}{V}\sum_{\alpha }\frac{{\overset{®}{R}}_{\alpha i}{\overset{®}{R}}_{\alpha j}}{R_{\alpha }^{2}}-P\delta_{ij}=3\nu k_{B}T\left(\frac{{\overset{®}{R}}_{\alpha i}{\overset{®}{R}}_{\alpha j}}{R_{\alpha }^{2}}\right)-P\delta_{ij}$$ 9

where *V* is the volume occupied by the network, **δ** is the Kronecker delta tensor, ν is the number density of phantom entropic strands between the cross-links, which may be either actual chemical cross-links or entanglements, and the indices *i* and *j* assume values *x*, *y* and *z*. For entropic strands, the natural (unstressed) length is zero. Therefore, without counteracting pressure *P*, a network of such strands would collapse. The stress tensor **σ** is the negative of the pressure tensor **P**, that is, **σ** = – **P**,^90^ and either tensor may be used in the literature. Here, we follow the standard convention in polymer physics and use the stress tensor **σ**,^7,8,29,44^ see, e.g., Section 9.2 of Doi’s book.^7^

It was shown by James and Guth^45–47^ that the mean end-to-end vectors of the strands of Gaussian networks displace affinely with the macroscopic deformation:

$${\overset{®}{R^{\prime }}}_{\alpha j}=E_{ij}{\overset{®}{R}}_{\alpha j}$$ 10

where ${\overset{®}{R^{\prime }}}_{\alpha }$ is the mean end-to-end vector of strand α after the deformation, **E** is the deformation tensor, and the summation rule is used for the indices that occur twice in a product.

Consider a homogeneous simple shear deformation in the *xy*-plane where a material point located at position **R** is displaced to position **R′**:

$$r_{x}^{\prime }=r_{x}+\gamma r_{y},r_{y}^{\prime }=r_{y},r_{z}^{\prime }=r_{z}$$ 11

where γ is the shear strain. The corresponding deformation tensor is given by

$$E_{ij}=\frac{\partial r_{i}^{\prime }}{\partial r_{j}}=\left(\begin{array}{lll} 1 & \gamma & 0\\ 0 & 1 & 0\\ 0 & 0 & 1 \end{array}\right)$$ 12

Using eq 12 in eq 10 one obtains

$${\overset{®}{R^{\prime }}}_{\alpha x}={\overset{®}{R}}_{\alpha x}+\gamma {\overset{®}{R}}_{\alpha y},\;{\overset{®}{R^{\prime }}}_{\alpha y}={\overset{-}{R}}_{\alpha y},\;{\overset{®}{R^{\prime }}}_{\alpha z}={\overset{-}{R}}_{\alpha z}$$ 13

The shear modulus *G* of an isotropic network is defined as

$$G=\underset{\gamma \to 0}{\lim}\left(\frac{1}{\gamma }\sigma_{xy}^{\prime }\right)$$ 14

where **σ′** is the stress tensor of the deformed network.

Substituting eq 13 into eq 9, and noting that for a macroscopically isotropic network the relations ⟨*R̅*_α*x*_ *R̅*_α*y*_⟩ = 0 and ⟨*R̅*_α*y*_^2^⟩ = ⟨**R̅**_α_^2^⟩ /3 hold, one finds:

$$\sigma_{xy}^{\prime }=3\nu k_{B}T\left\langle \frac{{\overset{®}{R^{\prime }}}_{\alpha x}{\overset{®}{R^{\prime }}}_{\alpha y}}{R_{\alpha }^{2}}\right\rangle =3\nu k_{B}T\gamma \left\langle \frac{{\overset{®}{R}}_{\alpha y}^{2}}{R_{\alpha }^{2}}\right\rangle =\nu k_{B}T\gamma \left\langle \frac{{\overset{®}{R}}_{\alpha }^{2}}{R_{\alpha }^{2}}\right\rangle$$ 15

Substituting eq 15 into eq 14 one obtains the same ANT formula [Disp-formula eq1], but now applicable to entangled polymer networks:

$$G_{\mathrm{ANT}}=\Gamma \nu k_{B}T\;\mathrm{with}\;\Gamma =\left\langle \frac{{\overset{®}{R}}_{\alpha }^{2}}{R_{\alpha }^{2}}\right\rangle$$ 16

where the network strands α may connect either actual chemical cross-links or entanglements, both of which are treated on an equal footing.

## 3 Methods: Force Balance Procedure

Our Force Balance procedure uses a Gaussian bead–spring representation of the polymer network. This procedure can be readily applied to existing network structures, such as those from coarse-grained Kremer-Grest (KG)^91,92^ MD simulations. This allows a direct comparison of the results for both validation and performance assessment. All computations and optimizations are performed in three dimensions, although, for clarity, most of the figures in this work show two-dimensional representations of the network structures.

However, using microstructures from MD simulations would be overall inefficient, as generating a network typically requires several CPU years of computation on a high-performance cluster,^49^ while the resulting network is ultimately utilized to determine the modulus via the Force Balance procedure in less than a minute on a personal computer. Therefore, it is appealing to achieve structure generation without the need for expensive MD simulations.

### 3.1 Removing Dependence on MD Simulations

A common approach to generate polymer networks using MD simulations is to start with a melt of polymer chains, to equilibrate this melt using the relevant force field with excluded volume nonbonded interactions, and then to cross-link the chains to form a network using a collision-diffusion procedure.^49,79,93^ In some cases, even the polymer melt chains are generated using such a collision-diffusion procedure starting from the monomers rather than a phantom melt.^94^ Due to the excluded volume interactions, particularly the equilibration step is computationally expensive, requiring several CPU years of computation on a high-performance cluster.^49^

There are alternative methods involving MD simulations that attempt to reduce this expense. For example, Zhang et al.^95^ proposed a method that sequentially backmaps coarse-grained configurations. Komarov et al.^96^ also apply a mapping, taking it even further by mapping between coarse-grained and atomistic representations of the polymer network. Forrest and Suter^97^ used time coarse-graining to accelerate the equilibration of polymer melts. Another approach involves prepacking the Gaussian chains to reduce the density fluctuations in the system, followed by a gradual introduction of the excluded volume interactions.^98^ One can also use a so-called double-bridging algorithm in which new bonds are formed across a pair of chains using MC steps, creating two new chains each of them different from the original ones.^98–100^ Nonetheless, all these methods still require some time-stepping or MD simulations, which we aim to avoid to reduce the computational cost of the structure generation.

In this work, we use an MC structure generation method.^42^ However, in contrast to previous publications that presented and used it,^42,48,101^ here, we require not only the connectivity between cross-links but also the positions of the internal beads of the strands, as those are needed for the sampling of entanglements as described in the next Section.

The generation of a polymer network using the MC method introduced in ref 42 starts by randomly placing the cross-links in a periodic box. Then, the following steps are repeated until either the target extent of reaction *p* or soluble fraction *w*_sol_ is reached: a free, unreacted strand end is selected randomly and if the other end of the selected strand is also free, a free cross-link site is chosen at random and the selected strand end is connected to the chosen cross-link site. However, if the opposite end of the selected strand is already connected to a cross-link, the cross-link site to connect the selected strand end to is chosen from the available free cross-link sites with a probability proportional to the radial Gaussian distance distribution function:

$$P(N,R)4\pi R^{2}dR=4\pi {\left(\frac{3}{2\pi Nb^{2}}\right)}^{3/2}\exp\left(-\frac{3R^{2}}{2Nb^{2}}\right)R^{2}dR$$ 17

where *R* is the distance between the chosen cross-link and the candidate site, *N* the number of bonds in the strand to be placed, and *b* the bond length.^29^

This results in a network of cross-links connected by both network and dangling and soluble strands (see Figure 1). A more detailed description of the procedure can be found in our previous works.^42,49,89^ In addition to this original MC method, the strands now need to be augmented with internal beads.

**Figure 1:**
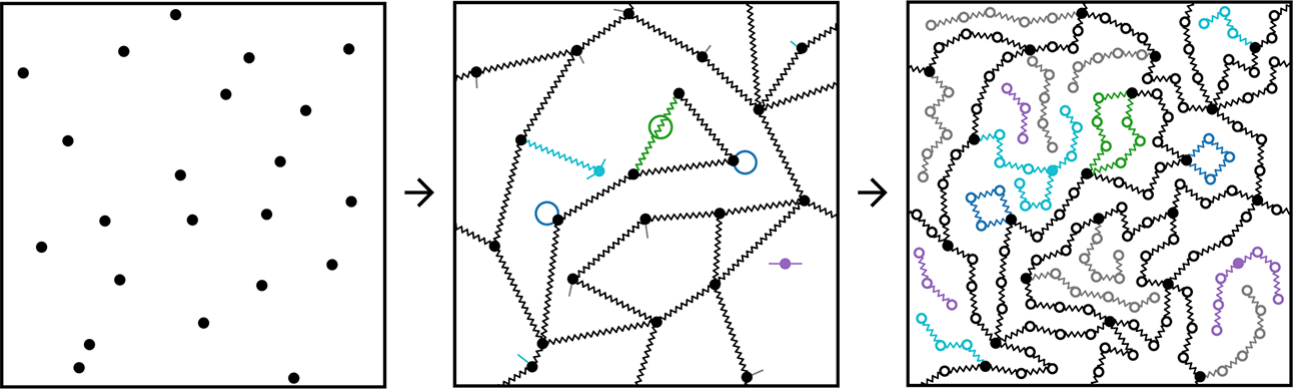
2D illustration of the MC structure generation method. First, cross-links (solid circles) are randomly distributed in a box. Then, strand ends are randomly connected to cross-links using eq 17. Primary (blue) and secondary (green) loops can already occur at this point. The short straight segments in the central panel indicate the dangling strands. Finally, beads are placed on the strands by applying a Brownian Bridge process, or just a random walk for monofunctional (gray), dangling (cyan), and free chains (purple).

We choose to use the standard Gaussian bead–spring chain model,^44^ where the bond between two connected beads is represented by a harmonic spring of natural length 0. For such a Gaussian chain, the relation ⟨*b* ^2^⟩ = α⟨*b*⟩^2^ with α = 3π/8 ≈ 1.178 holds, where *b* is the magnitude of the bond vector **b** between two connected beads. In order to utilize data from the existing literature to determine the appropriate number *N* of bonds to place between two cross-links, it is a sensible choice to use the Kuhn model as a foundation.^102^ Equating the contour length and the mean-square end-to-end distance of a freely joined Kuhn chain and the corresponding bead–spring chain gives, respectively,

$$N_{K}b_{K}=N\left\langle b\right\rangle \;\mathrm{and}\;N_{K}b_{K}^{2}=N\left\langle b^{2}\right\rangle$$ 18

where the subscript *K* stands for Kuhn segments. Therefore, we have

$$\left\langle b\right\rangle =b_{K}/\alpha \;\mathrm{and}\;N=\alpha N_{K}$$ 19

and correspondingly for the masses

$$NM=N_{K}M_{K}\Rightarrow M=M_{K}/\alpha$$ 20

For a particular polymer, the set of parameters needed can readily be determined from the parameters available for the Kuhn segments in the literature. For PDMS at *T* = 298 K one finds the values *b*_*K*_ = 1.142 nm and *M*_*K*_ = 309.28 g mol^–1^ in the literature,^103^ and hence, ⟨*b*⟩ = 0.969 nm and *M* = 262.52 g mol^–1^. For the network generation, apart from these polymer-specific parameters, one also needs the network-specific parameters, such as the stoichiometric imbalance *r*, the degree of cross-linking *p* (or, alternatively, the soluble fraction *w*_sol_), the molecular weight and mass fractions of the precursor chains, as well as the functionality *f* of the backbone or cross-link chains. A comprehensive list of the parameters of our model can be found in Table 1.

**Table 1:** Input Parameters for the MC Structure Generation and the Entanglement Sampling for the Reproduction of the Experimental Data Employed in This Work^a^

**symbol**	**input parameter**
*r*	stoichiometric imbalance
*f*	cross-link functionality
*p*	degree of polymerization
*b*_2_	fraction of active ends of *B*_2_ in a mixture of *B*_1_ and *B*_2_
*M*_*n*,*B*_2__	molecular weight of bifunctional chains
*M*_*n*,*B*_1__	molecular weight of monofunctional chains
*M*_*n*,*X*_	molecular weight of backbone/cross-link chain
*T*	reference temperature
*G*_e_(1)	entanglement modulus
*M*_*K*_	Kuhn mass
*l*_*K*_	Kuhn length
ρ	density
*c*_*s*_	entanglement sampling cutoff (fitting parameter)

a Some parameters are redundant, for example, the soluble fraction *w*_sol_ can be used instead of the degree of polymerization *p*, or the weight fractions of monofunctional and bifunctional chains instead of the coefficient *b*_2_. Additionally, one should also specify whether or not the soluble fraction is extracted before modulus measurement. Note that the entanglement sampling cutoff *c*_*s*_ is the only fitting parameter of our model.

For free and dangling chains, one can simply use a random walk to place their beads, since that will automatically result in the desired Gaussian distribution of end-to-end distances.^29^ Say we want to place *N* beads of a dangling chain, this random walk is performed for *N* steps starting at the cross-link to which the dangling chain is connected, and at each step, a new bead is placed and connected to the previously placed bead. For network strands, we apply a Brownian Bridge process:^104–108^ Suppose that we want to sample the *N* – 1 internal beads of a chain between the cross-links at positions **a** and **b**. For this, we generate a realization of a Brownian Bridge process, the set of coordinates **r**_*s*_ with *s* = 0, 1, ···, *N* and predefined **r**_0_ = **a** and **r**_*N*_ = **b**. We first perform an unconstrained random walk **R**_*s*_ with **R**_0_ = **a** and then obtain the bead coordinates as follows:

$$r_{s}=R_{s}-\frac{s}{N}\left(R_{N}-b\right)$$ 21

We have verified this placing procedure by asserting that neither the length, nor the end-to-end distance distribution of the bonds and strands, nor the modulus was affected by performing 10^5^ Metropolis MC steps for each internal bead.

This network generation method can be easily applied to more complicated topologies. For example, to generate bottlebrush or comb-like networks (see Figure 10), randomly functionalized backbone chains are generated by a random walk. Then, internal beads of the backbone chains are selected at random and converted to cross-links until the desired average functionality of the backbone chain is reached. The bifunctional cross-linker chains are placed, as usual, between two such backbone cross-links with the pair sampled according to the desired end-to-end distribution, e.g., eq 17.

### 3.2 Entanglement Representation

Recently, we have shown that for modeling viscoelastic properties, the entanglements can be introduced as additional springs.^102^ In this study, we further refine this model, by replacing the two beads of such an entanglement spring by a single entanglement bead, equivalent to a tetrafunctional cross-link (see Figure 2). This is consistent with the treatment of entanglements as tetrafunctional cross-links as found, for example, in the MMT.^57,109^

**Figure 2:**
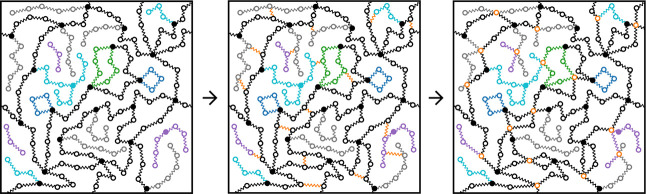
Illustration of the entanglement sampling procedure. Using a polymer network consisting of beads and springs forming chains, with both mono- (gray) and bifunctional strands (black), end-linked by tetrafunctional cross-links (black filled circles), pairs of beads are chosen randomly to form entangled pairs (orange springs, central panel). Then, each pair is collapsed to one entanglement bead (orange beads), which behaves like a tetrafunctional cross-link. The sketches also illustrate primary loops (blue), secondary loops (green), and soluble (purple) and dangling (cyan) structures.

#### 3.2.1 Entanglement Sampling

To sample the entanglements from a given bead–spring network, a bead in the structure is sampled at random (excluding cross-link beads and already created entanglement beads). Then, within a certain sampling cutoff *c*_*s*_, another bead is chosen randomly to represent the second end of the entanglement spring. These two beads are then merged into one single entanglement bead.

The entanglement density is obtained from the relation:^57^

$$G_{e}\left(1\right)=\epsilon k_{B}T$$ 22

where *G*_e_(1) is the entanglement modulus of a fully polymerized stoichiometric network, ϵ the corresponding entanglement density, *k*_B_ the Boltzmann constant, and *T* the temperature. For PDMS with an entanglement modulus from the literature of 0.25 MPa,^110^ at 298 K, an entanglement density of ϵ = 0.061 nm^–3^ results. However, the definition of the entanglement modulus is not unambiguous. Literature discusses its equality to the plateau modulus *G*_*N*_ of high molar mass polymer melts, with a measured value of 0.2 MPa,^103^ whereas modern theories of entangled polymer dynamics relate the plateau and entanglement modulus as $G_{e}\left(1\right)=\frac{5}{4}G_{N}$ ^44,111^ yielding 0.25 MPa.^110^ Recent MD simulations have suggested a value in between, namely 0.236 MPa.^89^ Following on from the theoretical studies, we will use 0.25 MPa for our procedure, except when comparing our predictions with the MD simulations mentioned.

The density of entanglements determines the number of entanglements to be sampled, whereas the sampling cutoff specifies the maximum permissible distance at which two randomly selected beads will be considered eligible for entanglement. This sampling cutoff value is the only adjustable parameter in our procedure. This parameter is expected to have one specific constant value for each particular type of polymer. A summary of all the parameters used in the MC structure generation and the entanglement sampling is given in Table 1.

#### 3.2.2 Removal of Inactive Entanglements

The initial entanglements are chosen regardless of the type of chain from which the sampled beads come from. Therefore, this process can also result in entanglements associated with dangling and soluble molecular structures (see Figure 3). However, for the calculation of the modulus, such structures and their associated entanglements should not be factored in. These structures are elastically ineffective and should collapse upon energy minimization, except for those involving catenated loops. Consequently, one needs to account for which of the four springs of an entanglement bead belongs to which strand, and then at regular intervals during the optimization Force Balance procedure one needs to remove entanglements associated with elastically inactive strands. Such strands can be identified simply by the collapsed springs between the cross-link and entanglement beads.

**Figure 3:**
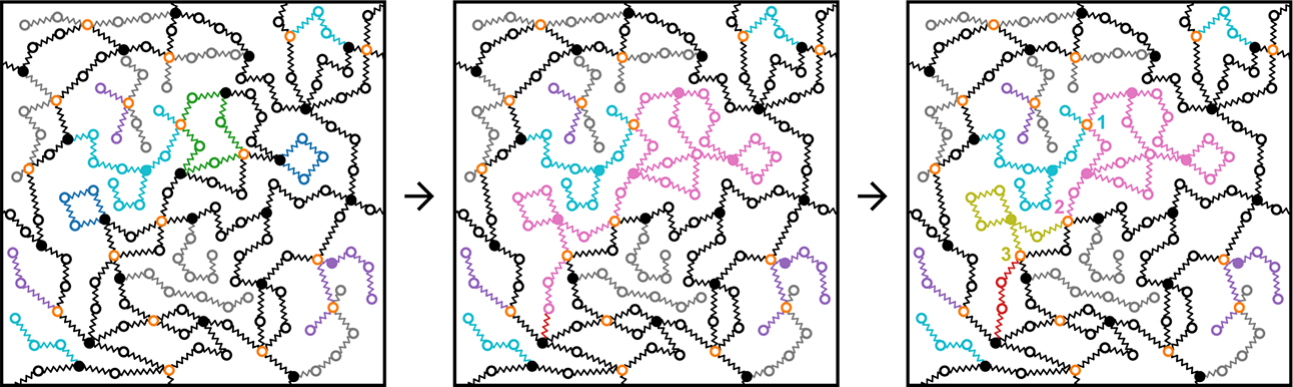
Exemplaric illustration of the need for the removal procedure. Simple algorithms cannot detect arbitrarily collapsible complex structures as the big one highlighted in pink in the central panel. The removal needs to run during several optimization steps since some structures can only become collapsing after some inactive entanglements have been removed, as highlighted in the third panel: The entanglement labeled 1 will be removed once the cyan dangling structure is collapsed. Thereafter, the pink structure can collapse. Once collapsed, the entanglement labeled 2 would be removed, whereafter the olive structure will collapse, the entanglement 3 be removed, and finally, the red part finishing the collapse of this elastically ineffective structure. Depending on the sampled entanglements, this structure could just as well have been elastically effective, for example, if the primary loops were entangled with the elastically effective part of the network.

In MD simulations, an increased proportion of elastically ineffective strands adversely affects the convergence of the simulation, as these strands introduce additional fluctuations that impact entropic quantities. On contrary, in the Force Balance procedure, the removal of nontrapped entanglements facilitates faster convergence in networks characterized by a high proportion of elastically inactive strands, since the number of degrees of freedom decreases.

### 3.3 Entanglement Sampling Cutoff

At this point, we still have a requirement for good reference data for the optimization of the single fitting parameter of our model, the entanglement sampling cutoff *c*_*s*_. This cutoff is defined as the maximum distance at which two beads can form an entanglement. MD data is an obvious choice for the reference data (see Figure 6), but would lead to another undesirable dependency on expensive MD simulations. Since we had previously shown^49,81^ that the MMT reproduces the modulus of end-linked networks formed from bulk as predicted by MD simulations, we decided to use the MMT as the reference standard to optimize the sampling cutoff. Contrary to our Force Balance procedure, the MMT has a few limitations, including the assumption that no loops are present. Consequently, we configure our entanglement sampling cutoff based on structures without primary or secondary loops, but then, in subsequent computations, conduct production measurements with structures that do have loops.

### 3.4 Optimization Procedure

Because the MC generation method does not impose any limitations on the structures, we can easily predict the modulus of more complicated systems, such as systems with monofunctional and bifunctional precursor chains and mixtures of cross-links with different functionalities. After generating the structure and sampling entanglements, the structure is optimized to its minimum energy state, and eq 16 is applied to calculate the modulus. No deformation is applied to obtain the modulus using the Force Balance procedure, as illustrated in Figure 4.

**Figure 4:**
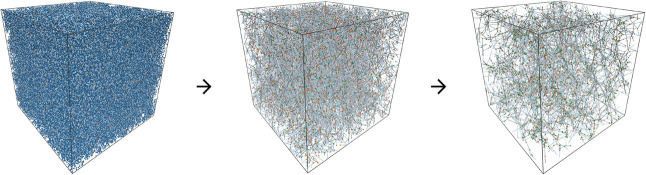
MC network with *f* = 4, *r* = 1, *p* = 0.8, and 10^4^ strands with 10 beads each. In the left panel, the internal beads of the strands are shown in blue, whereas the cross-links are shown in orange. In the central panel, the strands have been reduced to springs between the cross-links and entanglements (shown in green). The right panel shows the maximum entropy state of this network, with elastically inactive strands, entanglements, and cross-links omitted. In this microstructure, 58% of the strands are elastically effective.

To find the minimum energy state of the structure, a variety of methods can be chosen. Examples include iterative step methods, such as the steepest gradient descent, one in which the strand ends are displaced in steps according to the forces acting on them,^42^ or in which each strand end is placed in the position where all the forces acting on it cancel out.^112^ However, given the linear and sparse nature of the problem, we determined that the conjugate gradient method with a diagonal preconditioner is the most efficient strategy overall.^113,114^

## 4 Results and Discussion

For each set of input structural parameters, 10 structures were generated, for each of which the entanglements were sampled three times. Each structure consisted of approximately 10 000 chains, with small deviations coming from differences introduced by the functionality of the cross-links (for example, while a structure with *p* = 1, *r* = 1, and *f* = 4 cross-links may have 10 000 chains, the corresponding structure with *f* = 3 may have 9 999 chains). This structure size is consistent with the size used previously in MD simulations.^49,81,89^ See the Supporting Information (SI) for a Representative Volume Element (RVE) size study. All precursor chains are assumed to be monodisperse.

### 4.1 MMT vs Force Balance

To determine the entanglement sampling cutoff, the predictions of the Force Balance procedure are compared with those of the MMT. Figure 5a compares the MMT and Force Balance results for structures without primary or secondary loops, achieved by optimization for the best entanglement sampling cutoff. A value of 2.5 nm was found to be optimal, where all data points agree between the two methods within a margin of error of 10%. Notably, this value was found to be independent of the choice of the entanglement modulus within the three values studied 0.2 MPa,^103^ 0.236 MPa,^89^ and 0.25 MPa.^110^ This is a promising result, given that the phantom part of the moduli agrees no matter the choice, whereas the entanglement part has the entanglement modulus as its parameter for both MMT and the Force Balance.

**Figure 5:**
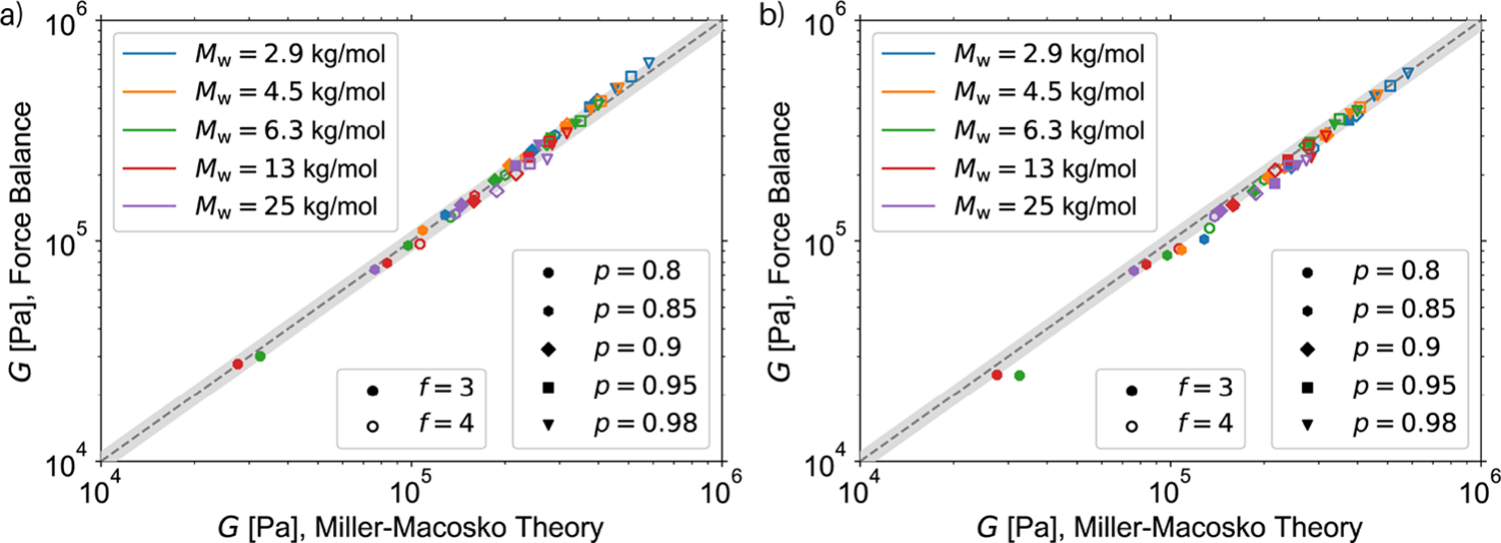
Modulus predicted by the MMT for different PDMS polymer networks with chains of different melt molar weight *M*_*w*_, degree of polymerization *p,* and cross-link functionality *f*, compared to the results from the Force Balance procedure. For all structures, a stoichiometric imbalance of *r* = 1 was used. The gray area indicates the ±10% deviation from perfect agreement. (a) Best agreement for structures without primary or secondary loops was found with an entanglement sampling cutoff *c*_*s*_ of 2.5 nm. (b) Using this optimal cutoff, one can assess the effect of the loops. It appears that the presence of loops is nearly negligible for higher degree of polymerization and longer chains.

In Figure 5b, the structures are generated as in Figure 5a, except that the formation of primary and secondary loops is not prevented. The comparison of the two subplots in Figure 5 shows that loop formation does have an influence, particularly for low degrees of polymerization and shorter chains. There are two main effects at play here: On the one hand, the probability of loop formation is higher for shorter chains whereas, and on the other hand, the probability that the formed loops are elastically active due to entanglements is higher for a higher degree of conversion. However, in all systems studied, the effect of loops is not significant enough to warrant a comparison with experimental results to assess the frequency of occurrence or effect of loops in the corresponding real experimental networks polymerized from bulk.

### 4.2 MC vs MD Structure Generation

In Figure 6a, the comparison is made between the data predicted from the MD stress-relaxation simulations^49^ and the corresponding predictions from the Force Balance procedure using the same MD structures. The MD structures were generated by equilibrating a melt of PDMS chains for 5 × 10^7^ steps and then cross-linking them using a collision-diffusion procedure and equilibrating the resulting network for another 5 × 10^7^ MD steps.^49^ The simulations were conducted in an *NVT* ensemble using a velocity-Verlet integrator with a time step of Δ*t* = 0.01τ and a Langevin thermostat with a damping constant of γ = 0.02/Δ*t*, where τ is the Lennard-Jones time unit, at a reference temperature of 298 K.^49^ For comparison, Figure 6b shows the predictions obtained using the corresponding MC-generated structures. It is apparent that the MC and MD generation procedures lead to slightly different predictions. The differences show similar trends when comparing the predictions obtained using MC generated structures with and without loops (see Figure 5). However, we have found that the loop fractions in the structures generated by MC and MD are comparable. These trends emerge from several other factors. On the one hand, the chains in the MD melts do not perfectly follow the linear relationship between the mean-square end-to-end distance and the number of FENE bonds for the shorter chains.^49,102^ On the other hand, by construction, the MC generator is guaranteed to generate microstructures with the end-to-end distances following the equilibrium distribution as in the melt, whereas the MD collision-diffusion generator usually does not maintain the equilibrium distribution and it reaches the desired degrees of polymerization already after less than 10 ns. This is much faster than what is common in the laboratory, where it can take multiple days to reach the desired degree of convergence.^86^

**Figure 6:**
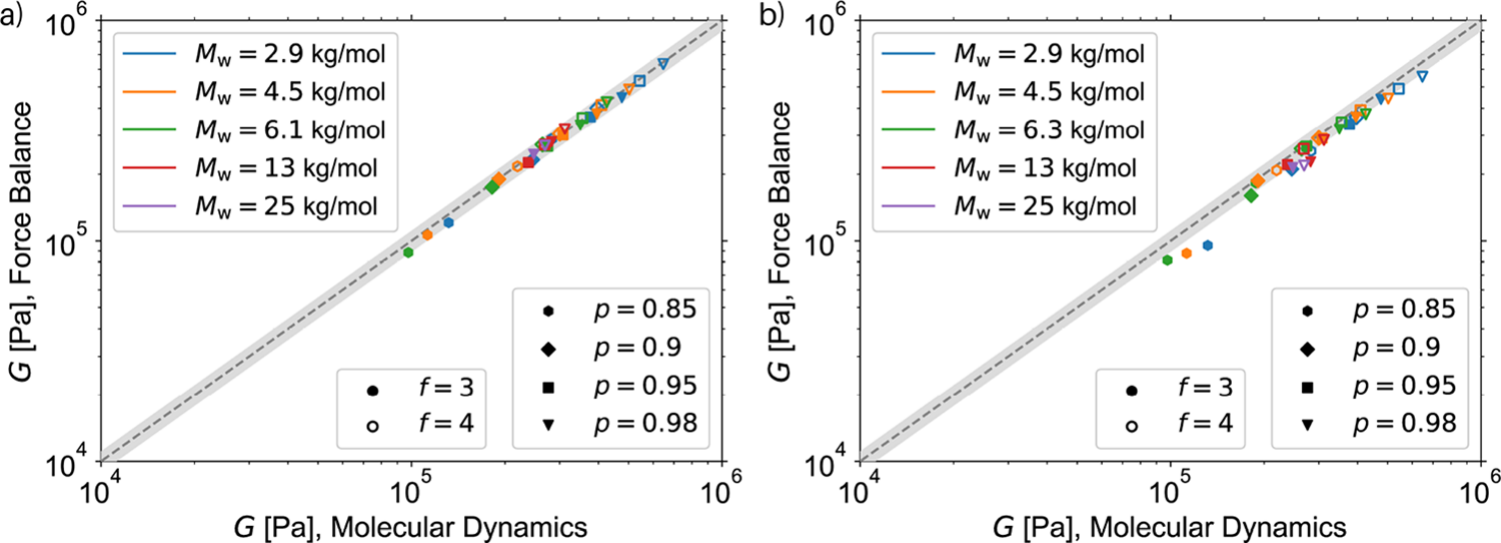
Comparison of the modulus as predicted by MD simulations^49^ and the Force Balance procedure for different PDMS polymer networks with different precursor molar weight *M*_*w*_, degree of polymerization *p,* and cross-link functionality *f*. (a) Same structures are used for the Force Balance as in MD simulations. (b) Structures are generated using the MC generation method. For all structures, a stoichiometric imbalance of *r* = 1 was used. The parameters used for the entanglements introduced for the Force Balance is an assumed entanglement modulus of 0.236 MPa since this is the entanglement modulus predicted by these MD simulations,^49^ and a sampling cutoff of 2.5 nm. The gray area highlights the 10% deviation from the ideal result, i.e., identical outcomes from both methods. The computational time requirement for the MD results is more than 20 000 times higher, many weeks on a supercomputer, compared to less than a minute on a personal laptop using the Force Balance procedure. Note that some systems readily available by the Force Balance procedure could not be obtained by the stress–relaxation MD simulations, such as those with *p* = 0.85, *M*_*w*_ ≥ 13 kg mol^–1^.^49^

### 4.3 Force Balance vs Experimental Data

After validation of the Force Balance procedure by comparing the predictions with theoretical (MMT, Figure 5) and simulation results (MD, Figure 6), it is natural and interesting to also compare its predictions with experimentally measured shear moduli. To be consistent, we only consider PDMS networks. We first discuss some general observations and procedures, after which we will list all the literature works we reproduced together with some caveats and experimental details that needed to be addressed. A compilation of all results is presented in Figure 9.

Unless otherwise specified, we adopt the reported values of the soluble weight fraction as the reference for the stopping criterion of the MC polymerization. We run the MC structure generator until the reported soluble fraction is reached as measured by using a force relaxation procedure assuming phantom chains. The soluble fraction is defined as the weight fraction of the strands and cross-links that are not connected to the elastically effective network. The elastically effective network is identified by the strands that have not collapsed after energy minimization. When the soluble fraction is removed before performing experimental measurements of the modulus, we follow this procedure accordingly and remove the soluble fraction as determined using the force relaxation procedure using phantom chains. Note that while our procedure now allows one to determine the soluble fraction while accounting for entanglement effects, the running soluble fraction during polymerization is still determined under the assumption of a phantom system without entanglements. This is due to various reasons, including the inability to consistently sample the same entanglements upon increasing the degree of polymerization, but most importantly because of the realization that the effect on the soluble fraction is minor and insignificant, as demonstrated in Figure 7. This can be explained by the absence of larger soluble structures that could catenate to the elastically effective network.

**Figure 7:**
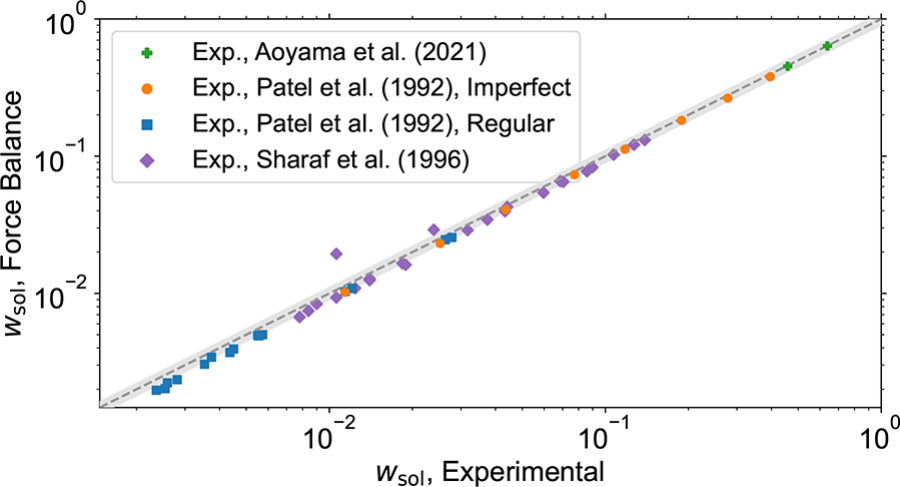
Horizontal axis lists experimental soluble fractions found in refs 88, 86, 85 . These values were used as a stopping criterion for our MC procedure to generate structures. For this Figure, the stopping criterion is the phantom soluble fraction being equal to the soluble fraction reported experimentally. The *y*-axis lists the soluble fraction as determined using the entangled Force Balance procedure. The reduction in soluble fraction due to introduction of entanglements is in all cases less than 20% compared to the procedure without entanglements (which shows perfect agreement by construction). We have not managed to physically reach the low *w*_sol_ reported for two outliers in the data from Sharaf et al. during cross-linking; however, since their *G* was measured after removal of the soluble fraction, we have still included these two points in Figure 9. The gray area highlights the 10% deviation from the ideal results, i.e., perfect agreement between predictions of the Force Balance procedure and experiments.

Reproducing experimental data can probably never be done exactly. Reasons include experimental uncertainties that can hardly be quantified. For example, if a measurement is conducted in a wet sample, either a second sample is required to determine the soluble fraction of the sample (and it is very difficult to obtain two exact same systems experimentally), or the sample may have lost some of the soluble fraction during the measurement.

When replicating the data, careful attention must be paid to the solvent, as it significantly impacts the results as to whether the polymerization was conducted with a solvent and whether the sample was dried prior to assessing the modulus. Herein, we aim to adhere to the synthesis procedures as accurately as possible.

Another difference between the literature experimental studies is the method used to obtain the modulus: Some studies use uniaxial elongation experiments, while others use shear experiments. In the uniaxial elongation experiments, the modulus may be further obtained in at least two ways. One can measure the slope of the tensile stress–strain curve at small strains, resulting in Young’s modulus *E*, which for incompressible isotropic materials is related to the shear modulus as *E* = 3*G*.^29^ Alternatively, one can measure the stress at a certain stretch ratio λ = *L*_1_/*L*_0_, where *L*_1_ is the length after elongation and *L*_0_ is the original length of the specimen. The modulus is then obtained as *G* = σ_*xx*_/(λ^2^ – 1/λ), where σ_*xx*_ is the stress in the direction of the elongation.^29^ However, our Force Balance procedure directly predicts the modulus *G* without any deformation using the ANT formula [Disp-formula eq16].

Among the data documented in the literature, we encountered three accounts (Chambon and Winter, 1987; Patel et al., 1992; Larsen et al., 2003) that pointed out challenges related to polymerization chemistry^84,85,87^ : The experimentalists report finding the highest modulus for a variable optimal stoichiometric imbalance *r* substantially higher than the expected *r* = 1. For these cases, we adapt the strategy of Larsen et al.^87^ They observed in their MC simulations that scaling the stoichiometric imbalance by the optimal value enables reproducing the measurements by their simulations. Our results also confirm this observation for all three of these examples, as the predictions agree surprisingly well with the measurements (see Figure 8). This is consistent in the sense that we reproduce the highest modulus obtainable for the given ratio of monofunctional to bifunctional precursor chains and their molecular weight. Even though this confirms that in all three examples the authors managed to achieve the highest modulus as intended, we recommend being cautious with this particular data, since quantification of the experimental artifacts is neither available nor possible. The agreement we see could just as well be coincidental, since the experimental results may be questioned for reasons that will be elucidated separately for each of the publications.

**Figure 8:**
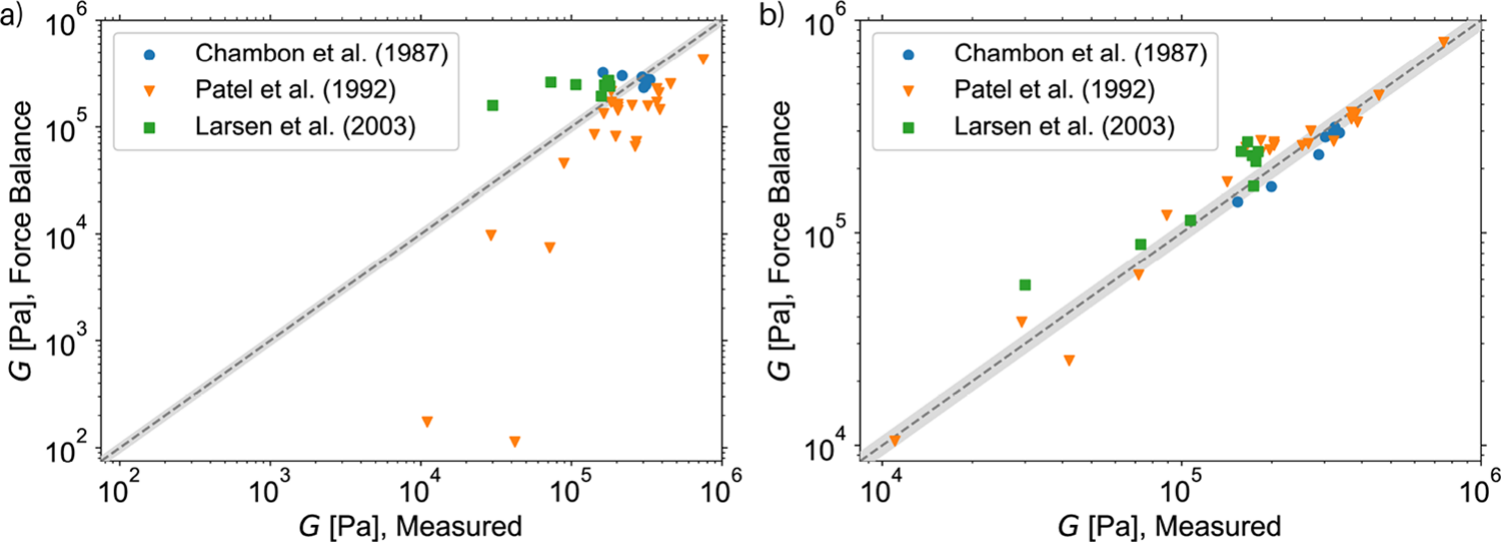
Comparison of measurements from the literature and predictions of the Force Balance procedure for the networks with the maximum modulus found at a stoichiometric imbalance substantially larger than 1. (a) Force Balance procedure is employed directly using the reported *r*. (b) *r* = 1 is used. The gray area highlights the 10% deviation from the ideal result, i.e., from a perfect prediction.

In the following short reviews of the literature, we will discuss all experimental procedures and our attempts to reproduce them using the Force Balance procedure, chronologically listed according to the publication date.

#### Gottlieb et al. (1981)

This article presents a large collection of data points from a variety of other publications.^82^ All polymers were synthesized in bulk. In all original publications available to us, the modulus was measured using uniaxial elongation experiments on dried samples.^115–122^ Note that here we have omitted data with a functionality higher than 4. Such structures will be discussed below together with more recent related data. Gottlieb et al. discuss possible issues with the measurements, such as inaccuracies in the measurement of the soluble fraction due to 2–5 wt % impurities in the commercial oligomeric PDMS reactants. The mean relative percentage deviation for these predictions is 30%.

#### Oppermann and Rennar (1987)

Uniaxial deformation experiments on circular symmetric dumbbell-shaped specimens were used to measure the modulus.^83^ Cross-linking was performed in bulk and the samples were slowly extracted, then deswollen and dried in vacuum, with a resulting weight loss of less than 1.5%. The scatter between the predictions and the measurements for these two data sets is significant, not least because of the age of the source, and yet the agreement is good.

#### Chambon and Winter (1987)

This is the first of the three examples where a stoichiometric imbalance of *r* > 1 was used to achieve the highest modulus.^84^ The samples were synthesized in bulk and the modulus of the networks was measured without extraction of the soluble fraction in oscillatory experiments at a low frequency of 0.5 rad s^–1^ and a small strain amplitude γ = 0.01. Note that for such high moduli, a significant error may be introduced by the compliance of the rheometer, which was not corrected for. However, this does not influence the ratios of the modulus measurements, which means that the optimal stoichiometric imbalance found would still lead to the highest modulus. Nevertheless, this may be an alternative explanation for why the agreement we see may be coincidental.

#### Patel et al. (1992)

This publication was influential, pushing the idea of adjusting the stoichiometric imbalance and using monofunctional chains to manipulate the stiffness and thus solving the technological problem of obtaining networks with low moduli.^85^ The experimentalists generated two different types of networks: model networks with a high modulus using bifunctional precursor chains, and imperfect networks using mixtures of monofunctional and bifunctional chains resulting in networks with lower moduli. This is the second example where the highest modulus was found for structures with a stoichiometric imbalance *r* > 1. Due to experimental artifacts, they found the highest modulus at a stoichiometric imbalance of up to *r* ≈ 1.7. The reasons for this deviation from the expected *r* = 1 have been reported to be possibly caused by steric hindrance, resulting in unequal reactivity of reactive sites of tetrafunctional cross-links, by side reactions that consume the silane hydrogens of the cross-links, or by additional vinyl groups being present in the precursor chains.^85^ Note that this optimal imbalance was determined by swelling experiments, whereas the reported moduli were obtained using oscillatory shear with parallel plate geometries. The measurements were performed without extracting the soluble fraction, and the weight fractions were determined afterward.

#### Sharaf et al. (1996)

A few years after the work by Patel et al., by rectifying the chemistry and thanks to advances in analytical methods and chemistry, Sharaf et al.^86^ managed to obtain their highest modulus (measured by uniaxial elongation experiments) close to the stoichiometric condition. This allows us to reproduce their data without the need for any compensation for the experimental uncertainties. The modulus was measured on samples that were extracted, deswollen, and finally dried under vacuum. For these data, good agreement is obtained between the measured moduli and the predictions from the Force Balance procedure (see Figure 9).

**Figure 9:**
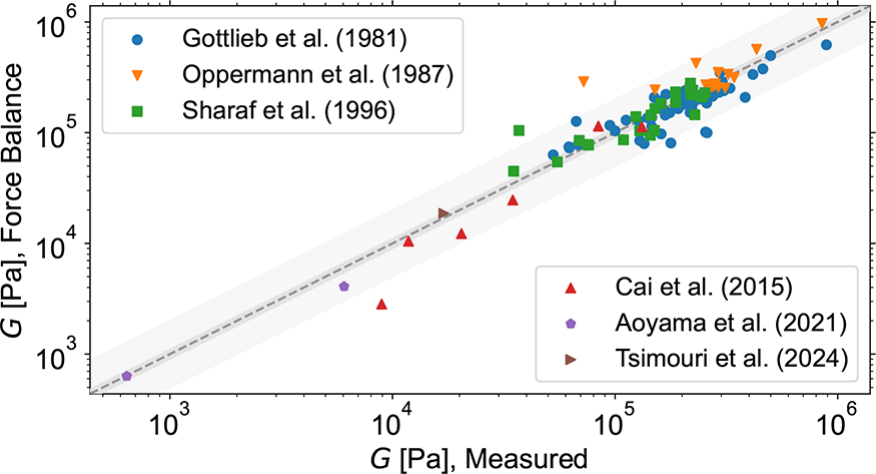
Comparison of the experimental modulus from refs 24,80,82,83,86,88 with the predictions of our Force Balance procedure using a sampling cutoff of 2.5 nm and an entanglement modulus of 0.25 MPa.^110^ Good agreement is found over a large variety of different structures. The darker gray area indicates the 10% deviation from perfect agreement, whereas the lighter one indicates a deviation by a factor of 2.

#### Larsen et al. (2003)

This is the third example of finding an optimal stoichiometric imbalance of *r* > 1.^87^ They just refer to the literature to explain the observed behavior, both to Patel et al.^85^ seeing this phenomenon caused by the maximum weight fraction of elastically effective chains, as well as to other works emphasizing the role of side reactions.^82,123,124^ The samples were generated in bulk and dried under vacuum. Measurements were performed using a custom-made filament stretch rheometer, at a constant Hencky strain rate of 10^–3^ s^–1^. Larsen et al. reproduced their measurements using Monte Carlo simulations, in which they scaled the stoichiometric imbalance by the optimal value (*r* ≈ 1.4) that they identified experimentally.

#### Cai et al. (2015)

All systems discussed this far could be reproduced just as well using the MMT.^80^ However, that would not result in equally good predictions and would also include certain assumptions such as the absence of loops. Our Force Balance procedure does not impose any limitations on the network architecture. This is why we can also apply it to more complex architecture networks like the ones studied by Cai et al.: The authors synthesized bottlebrush polymer networks (see Figure 10). This data set presents another minor difficulty: The authors reported a nonzero modulus already for precursor melts. They explain it by the side chains not being monofunctional but partially bifunctional. We compensate for this by assuming that a fraction of 2.6% of the monofunctional chains is bifunctional, as they proposed. Additionally, we compensate for steric effects due to the low density of the backbone chain and the side-chain length compared to that of the cross-linker chain length by preventing the cross-linker chains from connecting to the same backbone chain again. This is consistent with the sketch provided by Cai et al. in their Figure 1A. An alternative explanation for the lower predicted modulus without this compensation could be that certain entanglements are actually trapped due to steric effects.

**Figure 10:**
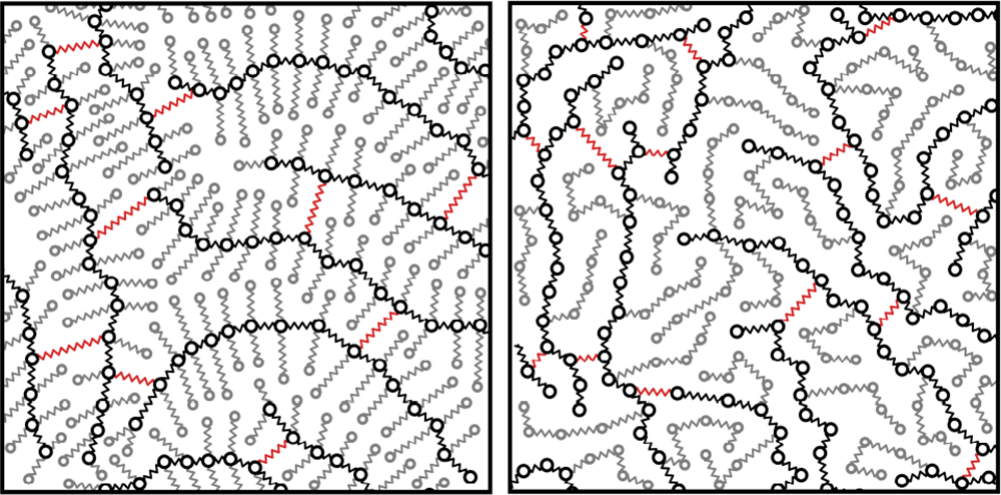
Illustration of a bead–spring model of a bottlebrush (left) and a comb-like (right) polymer network. Randomly functionalized backbone chains (black) have a number of attached monofunctional side chains (gray) and are cross-linked by bifunctional cross-linker chains (red).

#### Aoyama et al. (2021)

To investigate the applicability of our procedure to networks with very low moduli, we also reproduce the shear modulus of the near-critical gels reported by Aoyama et al.^88^ In this study, we adhere to the parameters (stoichiometric imbalance and target soluble fraction) outlined for the synthesis of their PDMS networks. They conducted measurements using stress-controlled rheometers with an oscillatory strain amplitude of 1% in the linear response range and an angular frequency between 0.1 and 100 s^–1^. The soluble fraction was not extracted before measurements. Such near-critical networks would require immense computing resources to study using MD simulations. For systems with 10^4^ chains, we estimate more than 10^3^ years of CPU time. Using our procedure, the systems are generated and analyzed in minutes on a personal computer. The agreement is good, given the higher sensitivity with regard to the input parameters such as stoichiometric imbalance and soluble fraction.

#### Tsimouri et al. (2024)

The authors present a novel approach to the fabrication of lightweight silicon and glass composites with submicrometer viscoelastic interlayers, achieving an exceptional balance between stiffness and damping.^24^ They used a network of comb-like polymers (see Figure 10). The modulus measurement was carried out using oscillatory shear at 1 Hz. The computational prediction is surprisingly good, with a relative error of about 5%.

Overall, as shown in Figure 9, the predictions of the presented Force Balance procedure agree quite well with the experimental results for all the networks studied, over 3 orders of magnitude of their equilibrium shear modulus *G*. However, there is a considerable amount of scatter, which also has to be attributed to experimental uncertainties as discussed in previous publications.^49,81^ Using both the experimental and MD results, we obtain a coefficient of determination *R* ^2^ of 0.851 and a mean relative percentage error of 19% (assuming that the experimental data are true).

## 5 Conclusions

A novel Monte Carlo procedure for generating computer microstructures of entangled Gaussian polymer networks is presented. A phantom Force Balance, Maximum Entropy Homogenization Procedure is introduced for entangled polymer networks and used to predict the equilibrium shear modulus of the microstructures while accounting for dangling and soluble structures, loops, and entanglements. The procedure gives predictions comparable in accuracy to molecular dynamics simulations, but while molecular dynamics simulations require many CPU years on high-performance computing clusters, our procedure gives predictions within less than a minute per structure without parallelization on an Apple M3 Pro MacBook for systems with 10^4^ chains from structure generation to the final result.

The proposed Force Balance procedure accounts for any topological imperfections and irregularities in the structure, including soluble chains, dangling strands, loops, and even entangled loops. Additionally, various topological parameters can be obtained using our procedure that are not immediately available from MD simulations, such as the fractions and compositions of branched soluble and dangling structures and entangled loops. All in all, our Force Balance procedure can be used practically for fast molecular prototyping and the design of polymer networks, over a wide range of technologically relevant equilibrium shear modulus values.

In our previous work,^102^ we proposed a normal mode approach to obtain the viscoelastic moduli of entangled Gaussian polymer networks. It will be interesting to see if and how this approach may be combined with the proposed Force Balance procedure in order to study the viscoelastic properties of complex architecture polymer networks.

Our presented Force Balance, Maximum Entropy Homogenization Procedure enables remarkably fast predictions of the equilibrium shear modulus of entangled polymer networks using the ANT formula [Disp-formula eq16], thereby allowing practical computational molecular-level design of complex-architecture networks with application-tailored stiffness. Extending the procedure to large-strain deformations and using the predicted stress–strain curves to extract parameters of the constitutive equations for continuum mechanics modeling of deformations and fracture of polymer networks, see, for example, Yang et al.^125^ and the references therein, is an interesting direction for future work. Compared to constitutive modeling, discrete mesoscopic models provide deeper insight into the structure and dynamics of polymer networks. For example, Wagner and Silberstein^126^ recently presented such a framework for large-strain time-dependent deformations of unentangled polymer networks and gels. Assadi et al.^127^ introduced a promising discrete network model to simulate the time-dependent mechanical properties of entangled polymer networks, where the entanglements were represented as sliding cross-linking junctions. It is worth exploring whether, and how, such dynamic entanglement modeling can be integrated into our framework to capture the viscoelastic behavior of entangled polymer networks. Recently, a simulation framework that combines our Monte Carlo network-generation algorithm with the kinetic theory of fracture was employed by Arora^101^ to predict the shear modulus and fracture strength of heterogeneous unentangled polymer networks. Incorporating their modified Monte Carlo generator for heterogeneous cross-link placement into our framework may offer a route to investigate how structural heterogeneity influences the modulus of entangled polymer networks.

## Supplementary Material



## Notes

Justification for the selected number of chains and MC realizations (PDF)

## References

[1] Hurley P. E. (1981). History
of Natural Rubber. Journal of Macromolecular
Science: Part A - Chemistry.

[2] Ji L. N. (2013). Study on
Preparation Process and Properties of Polyethylene Terephthalate (PET). Applied Mechanics and Materials.

[3] Rus D., Tolley M. T. (2015). Design, fabrication
and control of soft robots. Nature.

[4] Rogers J. A., Someya T., Huang Y. (2010). Materials
and mechanics for stretchable
electronics. Science.

[5] Foy J., Li Q., Goujon A., Colard-Itté J.-R., Fuks G., Moulin E., Schiffmann O., Dattler D., Funeriu D., Giuseppone N. (2017). Dual-light
control of nanomachines that integrate motor and modulator subunits. Nat. Nanotechnol..

[6] Li Q., Fuks G., Moulin E., Maaloum M., Rawiso M., Kulic I., Foy J. T., Giuseppone N. (2015). Macroscopic
contraction of a gel induced by the integrated motion of light-driven
molecular motors. Nat. Nanotechnol..

[7] Doi, M. Soft Matter Physics, 1st ed.; Oxford University Press: Oxford; New York, 2013.

[8] Doi, M. Introduction to Polymer Physics, reprint ed.; Oxford science publications; Clarendon Press: Oxford, 2010.

[9] Bishop, D. C. ; Johnson, R. E. D. The Mechanics of Vibration; Cambridge University Press, 1960.

[10] Christensen, R. M. Theory of Viscoelasticity: an Introduction; Academic Press: New York, 1971.

[11] Ferry, J. D. Viscoelastic Properties of Polymers, 3rd ed.; John Wiley & Sons: New York Chichester Brisbane Toronto Singapore, 1980.

[12] Jones, D. I. G. Handbook of Viscoelastic Vibration Damping; John Wiley & Sons, 2001.

[13] Lakes, R. Viscoelastic Materials; Cambridge University Press: Cambridge, 2009.

[14] Mead, D. J. Passive Vibration Control; Wiley, 1998.

[15] Nashif, A. D. ; Jones, D. I. G. ; Henderson, J. P. Vibration Damping; John Wiley & Sons, 1991.

[16] Rao M. D. (2003). Recent
applications of viscoelastic damping for noise control in automobiles
and commercial airplanes. Journal of Sound and
Vibration.

[17] Chen C. P., Lakes R. S. (1993). Viscoelastic behaviour of composite materials with
conventional- or negative-Poisson’s-ratio foam as one phase. J. Mater. Sci..

[18] Chen C. P., Lakes R. S. (1993). Analysis of high-loss viscoelastic composites. J. Mater. Sci..

[19] Brodt M., Lakes R. S. (1995). Composite Materials Which Exhibit High Stiffness and
High Viscoelastic Damping. Journal of Composite
Materials.

[20] Lakes R. S. (2002). High Damping
Composite Materials: Effect of Structural Hierarchy. Journal of Composite Materials.

[21] Unwin A. P., Hine P. J., Ward I. M., Fujita M., Tanaka E., Gusev A. A. (2018). Escaping the Ashby
limit for mechanical damping/stiffness
trade-off using a constrained high internal friction interfacial layer. Sci. Rep..

[22] Unwin A. P., Hine P. J., Ward I. M., Fujita M., Tanaka E., Gusev A. A. (2018). Novel phase separated
multi-phase materials combining
high viscoelastic loss and high stiffness. Compos.
Sci. Technol..

[23] Tsimouri I. C., Montibeller S., Kern L., Hine P. J., Spolenak R., Gusev A. A., Danzi S. (2021). A simulation-driven design approach
to the manufacturing of stiff composites with high viscoelastic damping. Compos. Sci. Technol..

[24] Tsimouri I. C., Caseri W., Hine P. J., Gusev A. A. (2024). Lightweight silicon
and glass composites with submicron viscoelastic interlayers and unconventional
combinations of stiffness and damping. Composites
Part B: Engineering.

[25] Sun J.-Y., Zhao X., Illeperuma W. R. K., Chaudhuri O., Oh K. H., Mooney D. J., Vlassak J. J., Suo Z. (2012). Highly stretchable
and tough hydrogels. Nature.

[26] Hoare T. R., Kohane D. S. (2008). Hydrogels in drug
delivery: Progress and challenges. Polymer.

[27] Dragan E. S. (2014). Design
and applications of interpenetrating polymer network hydrogels. A review. Chemical Engineering Journal.

[28] Langer R., Tirrell D. A. (2004). Designing materials
for biology and medicine. Nature.

[29] Rubinstein, M. ; Colby, R. H. Polymer Physics, 1st ed.; Oxford University Press: Oxford; New York, 2003.

[30] Reynolds V. G., Mukherjee S., Xie R., Levi A. E., Atassi A., Uchiyama T., Wang H., Chabinyc M. L., Bates C. M. (2020). Super-soft
solvent-free bottlebrush elastomers for touch sensing. Materials Horizons.

[31] Wilder E. A., Guo S., Lin-Gibson S., Fasolka M. J., Stafford C. M. (2006). Measuring the Modulus
of Soft Polymer Networks via a Buckling-Based Metrology. Macromolecules.

[32] Sheiko S. S., Dobrynin A. V. (2019). Architectural Code for Rubber Elasticity: From Supersoft
to Superfirm Materials. Macromolecules.

[33] Shokrani H., Shokrani A., Jouyandeh M., Seidi F., Gholami F., Kar S., Munir M. T., Kowalkowska-Zedler D., Zarrintaj P., Rabiee N., Saeb M. R. (2022). Green Polymer
Nanocomposites for
Skin Tissue Engineering. ACS Applied Bio Materials.

[34] Jeong K.-H., Park D., Lee Y.-C. (2017). Polymer-based
hydrogel scaffolds
for skin tissue engineering applications: a mini-review. J. Polym. Res..

[35] Kim T. G., Shin H., Lim D. W. (2012). Biomimetic
Scaffolds for Tissue Engineering. Adv. Funct.
Mater..

[36] Zhu J. (2012). Biomimetic
Hydrogels as Scaffolds for Tissue Engineering. J. Bioeng. Bioelectron..

[37] Chen G., Hou K., Yu N., Wei P., Chen T., Zhang C., Wang S., Liu H., Cao R., Zhu L., Hsiao B. S., Zhu M. (2022). Temperature-adaptive hydrogel optical
waveguide with soft tissue-affinity for thermal regulated interventional
photomedicine. Nat. Commun..

[38] Nizamoglu S., Gather M. C., Humar M., Choi M., Kim S., Kim K. S., Hahn S. K., Scarcelli G., Randolph M., Redmond R. W., Yun S. H. (2016). Bioabsorbable polymer
optical waveguides for deep-tissue photomedicine. Nat. Commun..

[39] Liechty W. B., Kryscio D. R., Slaughter B. V., Peppas N. A. (2010). Polymers for Drug
Delivery Systems. Annu. Rev. Chem. Biomol. Eng..

[40] Sung Y. K., Kim S. W. (2020). Recent advances
in polymeric drug delivery systems. Biomater.
Res..

[41] Ashby, M. Material property data for engineering materials, 2021; https://www.ansys.com/content/dam/amp/2021/august/webpage-requests/education-resources-dam-upload-batch-2/material-property-data-for-eng-materials-BOKENGEN21.pdf (accessed February 13, 2025).

[42] Gusev A. A. (2019). Numerical
Estimates of the Topological Effects in the Elasticity of Gaussian
Polymer Networks and Their Exact Theoretical Description. Macromolecules.

[43] Christensen, R. M. Theory of Viscoelasticity, 2nd ed.; Courier Corporation, 2013.

[44] Doi, M. ; Edwards, S. F. The Theory of Polymer Dynamics; International Series of Monographs on Physics; Oxford University Press: Oxford, NY, 1988.

[45] James H. M., Guth E. (1943). Theory of the Elastic Properties of Rubber. J. Chem. Phys..

[46] James H. M. (1947). Statistical
Properties of Networks of Flexible Chains. J.
Chem. Phys..

[47] James H. M., Guth E. (1947). Theory of the Increase
in Rigidity of Rubber during Cure. J. Chem.
Phys..

[48] Sen D., Olsen B. D. (2025). Quantification of
cyclic topology in polymer networks
using 3D nets. Physical Review Materials.

[49] Gusev A. A., Schwarz F. (2022). Molecular Dynamics
Study on the Validity of Miller–Macosko
Theory for Entanglement and Crosslink Contributions to the Elastic
Modulus of End-Linked Polymer Networks. Macromolecules.

[50] Kuhn W. (1936). Beziehungen
zwischen Molekülgröße, statistischer Molekülgestalt
und elastischen Eigenschaften hochpolymerer Stoffe. Kolloid-Z..

[51] Rubinstein M., Panyukov S. (1997). Nonaffine Deformation and Elasticity of Polymer Networks. Macromolecules.

[52] Rubinstein M., Panyukov S. (2002). Elasticity of Polymer Networks. Macromolecules.

[53] Panyukov S. (2020). Theory of
Flexible Polymer Networks: Elasticity and Heterogeneities. Polymers.

[54] Mark, J. E. ; Erman, B. Rubberlike Elasticity: A Molecular Primer, 2nd ed.; Cambridge University Press: Cambridge, 2007.

[55] Treloar, L. R. G. The physics of rubber elasticity, 3rd ed.; Oxford classic texts in the physical sciences; Oxford university press: New York, 2005.

[56] Flory P. J., Gordon M., Flory P. J., McCrum N. G. (1997). Statistical thermodynamics
of random networks. Proc. R. Soc. London, Ser.
A.

[57] Miller D. R., Macosko C. W. (1976). A New Derivation
of Post Gel Properties of Network
Polymers. Macromolecules.

[58] Lang M. (2018). Elasticity
of Phantom Model Networks with Cyclic Defects. ACS Macro Lett..

[59] Lang M. (2019). On the Elasticity
of Polymer Model Networks Containing Finite Loops. Macromolecules.

[60] Panyukov S. (2019). Loops in Polymer
Networks. Macromolecules.

[61] Zhong M., Wang R., Kawamoto K., Olsen B. D., Johnson J. A. (2016). Quantifying
the impact of molecular defects on polymer network elasticity. Science.

[62] Wang R., Alexander-Katz A., Johnson J. A., Olsen B. D. (2016). Universal Cyclic
Topology in Polymer Networks. Phys. Rev. Lett..

[63] Wang J., Lin T.-S., Gu Y., Wang R., Olsen B. D., Johnson J. A. (2018). Counting Secondary
Loops Is Required for Accurate Prediction
of End-Linked Polymer Network Elasticity. ACS
Macro Lett..

[64] Lin T.-S., Wang R., Johnson J. A., Olsen B. D. (2019). Revisiting the Elasticity
Theory for Real Gaussian Phantom Networks. Macromolecules.

[65] Busse W. F. (1932). The Physical
Structure of Elastic Colloids. J. Phys. Chem..

[66] Treloar L. R. G. (1940). Elastic
recovery and plastic flow in raw rubber. Trans.
Faraday Soc..

[67] Langley N. R. (1968). Elastically
Effective Strand Density in Polymer Networks. Macromolecules.

[68] Dossin L. M., Graessley W. W. (1979). Rubber Elasticity of Well-Characterized Polybutadiene
Networks. Macromolecules.

[69] Ball R., Doi M., Edwards S., Warner M. (1981). Elasticity of entangled networks. Polymer.

[70] Edwards S. F., Vilgis T. (1986). The effect of entanglements in rubber elasticity. Polymer.

[71] Edwards S. F., Vilgis T. A. (1988). The tube model theory of rubber elasticity. Rep. Prog. Phys..

[72] Kaliske M., Heinrich G. (1999). An Extended Tube-Model for Rubber
Elasticity: Statistical-Mechanical
Theory and Finite Element Implementation. Rubber
Chem. Technol..

[73] Heinrich G., Straube E. (1983). On the strength and deformation dependence of the tube-like
topological constraints of polymer networks, melts and concentrated
solutions. I. The polymer network case. Acta
Polym..

[74] Mergell B., Everaers R. (2001). Tube Models for Rubber-Elastic Systems. Macromolecules.

[75] Davidson J. D., Goulbourne N. C. (2013). A nonaffine network model for elastomers undergoing
finite deformations. Journal of the Mechanics
and Physics of Solids.

[76] Lang M. (2017). Relation between
Cross-Link Fluctuations and Elasticity in Entangled Polymer Networks. Macromolecules.

[77] Xiang Y., Zhong D., Wang P., Mao G., Yu H., Qu S. (2018). A general constitutive model of soft elastomers. Journal of the Mechanics and Physics of Solids.

[78] Xiang Y., Zhong D., Rudykh S., Zhou H., Qu S., Yang W. (2020). A Review of Physically
Based and Thermodynamically Based Constitutive
Models for Soft Materials. J. Appl. Mech..

[79] Gula I. A., Karimi-Varzaneh H. A., Svaneborg C. (2020). Computational Study of Cross-Link
and Entanglement Contributions to the Elastic Properties of Model
PDMS Networks. Macromolecules.

[80] Cai L.-H., Kodger T. E., Guerra R. E., Pegoraro A. F., Rubinstein M., Weitz D. A. (2015). Soft Poly­(dimethylsiloxane)
Elastomers from Architecture-Driven
Entanglement Free Design. Adv. Mater..

[81] Tsimouri I. C., Schwarz F., Bernhard T., Gusev A. A. (2024). A Comparison between
Predictions of the Miller–Macosko Theory, Estimates from Molecular
Dynamics Simulations, and Long-Standing Experimental Data of the Shear
Modulus of End-Linked Polymer Networks. Macromolecules.

[82] Gottlieb M., Macosko C. W., Benjamin G. S., Meyers K. O., Merrill E. W. (1981). Equilibrium
modulus of model poly­(dimethylsiloxane) networks. Macromolecules.

[83] Oppermann, W. ; Rennar, N. Permanent and Transient Networks; Progress in Colloid & Polymer Science; Steinkopff: Darmstadt, 1987; Vol. 75, pp 49–54.

[84] Chambon F., Winter H. H. (1987). Linear Viscoelasticity
at the Gel Point of a Crosslinking
PDMS with Imbalanced Stoichiometry. J. Rheol..

[85] Patel S. K., Malone S., Cohen C., Gillmor J. R., Colby R. H. (1992). Elastic
modulus and equilibrium swelling of poly­(dimethylsiloxane) networks. Macromolecules.

[86] Sharaf M. A., Mark J. E., Alshamsi A. S. (1996). Moduli of Elastomeric Networks Prepared
by Tetrafunctionally Endlinking Vinyl-Terminated Poly­(dimethylsiloxane)
Chains at Low Temperature. Polym. J..

[87] Larsen A. L., Hansen K., Sommer-Larsen P., Hassager O., Bach A., Ndoni S., Jo̷rgensen M. (2003). Elastic Properties
of Nonstoichiometric
Reacted PDMS Networks. Macromolecules.

[88] Aoyama T., Yamada N., Urayama K. (2021). Nonlinear Elasticity of Ultrasoft
Near-Critical Gels with Extremely Sparse Network Structures Revealed
by Biaxial Stretching. Macromolecules.

[89] Gusev A. A., Schwarz F. (2019). Molecular Dynamics
Validation and Applications of the
Maximum Entropy Homogenization Procedure for Predicting the Elastic
Properties of Gaussian Polymer Networks. Macromolecules.

[90] Shi K., Smith E. R., Santiso E. E., Gubbins K. E. (2023). A perspective on
the microscopic pressure (stress) tensor: History, current understanding,
and future challenges. J. Chem. Phys..

[91] Kremer K., Grest G. S. (1990). Dynamics of entangled linear polymer melts: A molecular-dynamics
simulation. J. Chem. Phys..

[92] Grest G. S., Kremer K. (1986). Molecular dynamics
simulation for polymers in the presence
of a heat bath. Phys. Rev. A.

[93] Svaneborg C., Everaers R., Grest G. S., Curro J. G. (2008). Connectivity and
Entanglement Stress Contributions in Strained Polymer Networks. Macromolecules.

[94] Doherty D. C., Holmes B. N., Leung P., Ross R. B. (1998). Polymerization molecular
dynamics simulations. I. Cross-linked atomistic models for poly­(methacrylate)
networks. Comput. Theor. Polym. Sci..

[95] Zhang G., Moreira L. A., Stuehn T., Daoulas K. C., Kremer K. (2014). Equilibration
of High Molecular Weight Polymer Melts: A Hierarchical Strategy. ACS Macro Lett..

[96] Komarov P. V., Yu-Tsung C., Shih-Ming C., Khalatur P. G., Reineker P. (2007). Highly Cross-Linked
Epoxy Resins: An Atomistic Molecular Dynamics Simulation Combined
with a Mapping/Reverse Mapping Procedure. Macromolecules.

[97] Forrest B. M., Suter U. W. (1995). Accelerated equilibration of polymer melts by time-coarse-graining. J. Chem. Phys..

[98] Auhl R., Everaers R., Grest G. S., Kremer K., Plimpton S. J. (2003). Equilibration
of long chain polymer melts in computer simulations. J. Chem. Phys..

[99] Pant P. V. K., Theodorou D. N. (1995). Variable Connectivity Method for the Atomistic Monte
Carlo Simulation of Polydisperse Polymer Melts. Macromolecules.

[100] Mavrantzas V. G., Boone T. D., Zervopoulou E., Theodorou D. N. (1999). End-Bridging Monte Carlo: A Fast Algorithm for Atomistic
Simulation of Condensed Phases of Long Polymer Chains. Macromolecules.

[101] Arora A. (2025). Effect of Spatial Heterogeneity on
the Elasticity and Fracture of
Polymer Networks. Macromolecules.

[102] Gusev A. A., Bernhard T. (2024). Molecular Model for
Linear Viscoelastic
Properties of Entangled Polymer Networks. Macromolecules.

[103] Fetters, L. J. ; Lohse, D. J. ; Colby, R. H. In Physical Properties of Polymers Handbook; Mark, J. E. , Ed.; Springer, New York: New York, NY, 2007; pp 447–454.

[104] Mansuy, R. ; Yor, M. , Eds., Aspects of Brownian Motion; Universitext; Springer: Berlin Heidelberg, 2008.

[105] Ibe, O. C. In Markov Processes for Stochastic Modeling, 2nd ed., Ibe, O. C. , Ed.; Elsevier: Oxford, 2013; pp 263–293.

[106] Chow W. C. (2009). Brownian bridge. WIREs Computational
Statistics.

[107] Martínez S., Petritis D. (1996). Thermodynamics of a
Brownian bridge
polymer model in a random environment. Journal
of Physics A: Mathematical and General.

[108] Wang S., Ramkrishna D., Narsimhan V. (2020). Exact sampling
of polymer conformations using Brownian bridges. J. Chem. Phys..

[109] Macosko C. W., Miller D. R. (1976). A New Derivation
of Average Molecular
Weights of Nonlinear Polymers. Macromolecules.

[110] Everaers R., Karimi-Varzaneh H. A., Fleck F., Hojdis N., Svaneborg C. (2020). Kremer–Grest
Models for Commodity Polymer Melts:
Linking Theory, Experiment, and Simulation at the Kuhn Scale. Macromolecules.

[111] Larson, R. G. The structure and rheology of complex fluids; Topics in chemical engineering; Oxford University Press: New York, 1999.

[112] Müller T., Sommer J.-U., Lang M. (2022). Elasticity of Tendomer
Gels. Macromolecules.

[113] Hestenes M., Stiefel E. (1952). Methods of conjugate
gradients for
solving linear systems. Journal of Research
of the National Bureau of Standards.

[114] Saad, Y. Iterative methods for sparse linear systems, 2nd ed.; SIAM: Philadelphia, 2003.

[115] Valles E.
M., Macosko C. W. (1979). Properties
of Networks Formed by
End Linking of Poly­(dimethylsiloxane). Macromolecules.

[116] Mark J. E., Sullivan J. L. (1977). Model networks of
end-linked polydimethylsiloxane
chains. I. Comparisons between experimental and theoretical values
of the elastic modulus and the equilibrium degree of swelling. J. Chem. Phys..

[117] Llorente M. A., Mark J. E. (1980). Model Networks of End-Linked Poly­(dimethylsiloxane)
Chains. 8. Networks Having Cross-Links of Very High Functionality. Macromolecules.

[118] Llorente M. A., Andrady A. L., Mark J. E. (1981). Model networks of
end-linked polydimethylsiloxane chains. Colloid
Polym. Sci..

[119] Meyers K. O., Bye M. L., Merrill E. W. (1980). Model Silicone Elastomer
Networks of High Juction Functionality: Synthesis, Tensile Behavior,
Swelling Behavior, and Comparison with Molecular Theories of Rubber
Elasticity. Macromolecules.

[120] Granick S., Pedersen S., Nelb G. W., Ferry J. D., Macosko C. W. (1981). Stress relaxation and dynamic viscoelastic
properties
of end-linked poly­(dimethyl siloxane) networks containing unattached
poly­(dimethyl siloxane). J. Polym. Sci., Polym.
Phys. Ed..

[121] Macosko C. W., Benjamin G. S. (1981). Modulus of three and four functional
poly­(dimethylsiloxane) networks. Pure Appl.
Chem..

[122] Mark J. E., Rahalkar R. R., Sullivan J. L. (1979). Model networks of
end-linked polydimethylsiloxane chains. III. Effect of the functionality
of the cross-links. J. Chem. Phys..

[123] Gent A. N., Tobias R. H. (1982). Diffusion and equilibrium
swelling
of macromolecular networks by their linear homologs. J. Polym. Sci., Polym. Phys. Ed..

[124] Sharaf M. A., Mark J. E., Ahmed E. (1994). Elastomeric
properties
of end-linked networks of high cross-link functionality. Accounting
for possible changes in effective functionality with extent of reaction
and chain-length distribution. Colloid &
Polymer Science.

[125] Yang J., Chen K., Yu C., Zhou K., Kang G. (2025). A hyperelastic
constitutive model for soft elastomers considering
the entanglement-dependent finite extensibility. Journal of the Mechanics and Physics of Solids.

[126] Wagner R. J., Silberstein M. N. (2025). A foundational
framework for the
mesoscale modeling of dynamic elastomers and gels. Journal of the Mechanics and Physics of Solids.

[127] Assadi S., Lamont S. C., Hansoge N., Liu Z., Crespo-Cuevas V., Salmon F., Vernerey F. J. (2025). Nonaffine
motion
and network reorganization in entangled polymer networks. Soft Matter.

