# Supplementary material for: Phantom Force Balance Procedure for Predicting the Modulus of Entangled Polymer Networks
Source: ACS Polym Au. 2025 Aug 5;5(5):500–13. doi: 10.1021/acspolymersau.5c00036 (PMC12511982; doi:10.1021/acspolymersau.5c00036)
Supplement: Supplementary file 1 [file lg5c00036_si_001.pdf]

Supporting Information for

Phantom Force Balance Procedure for Predicting the Modulus of Entangled Polymer Networks

Tim Bernhard[*,†,‡] and Andrei A. Gusev[*,‡]

†*Laboratory for Nanometallurgy, Department of Materials, ETH Zürich, 8093 Zürich, Switzerland*

‡*Department of Materials, ETH Zürich, 8093 Zürich, Switzerland*

E-mail: tim.bernhard@mat.ethz.ch; gusev@mat.ethz.ch

This supporting information provides justification for the selected number of chains and MC realizations. For the production computations reported in this work, we have used ten MC realizations with at least 10^4 chains each.

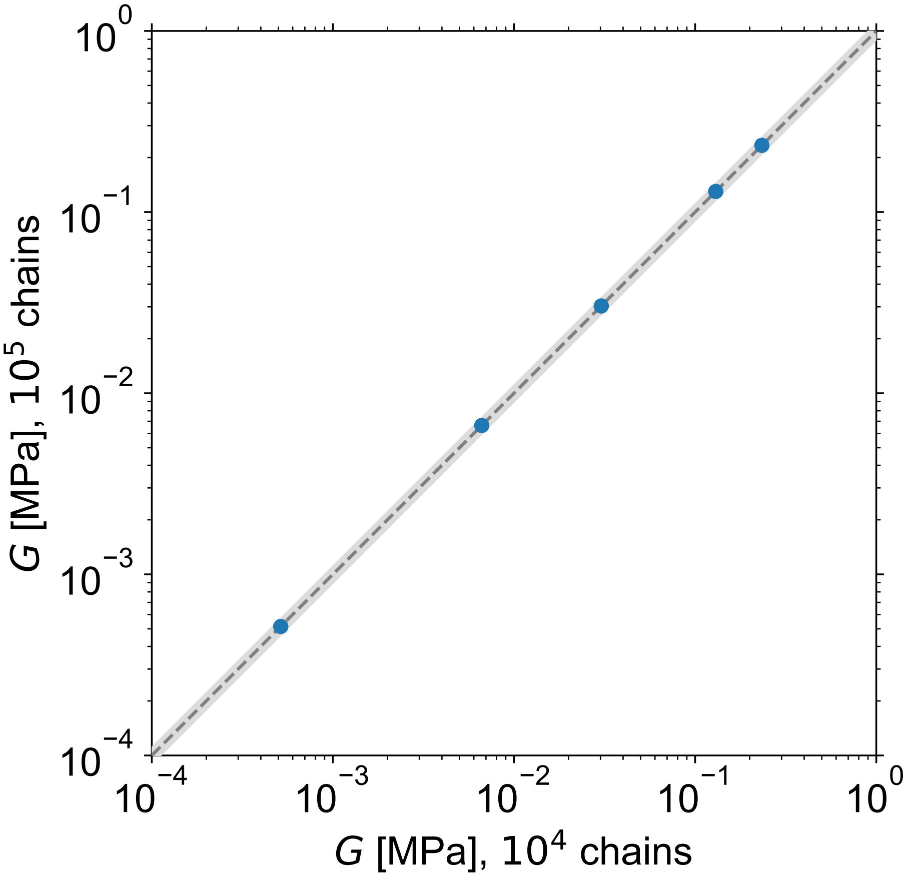

Figure S1: Effect of the system size on the equilibrium shear modulus G predicted using the Force Balance procedure. The averages over ten different MC realizations are shown. The grey band indicates the 10 % deviation from the perfect agreement (dashed diagonal line). The input parameters for the systems shown are listed in Table S1.

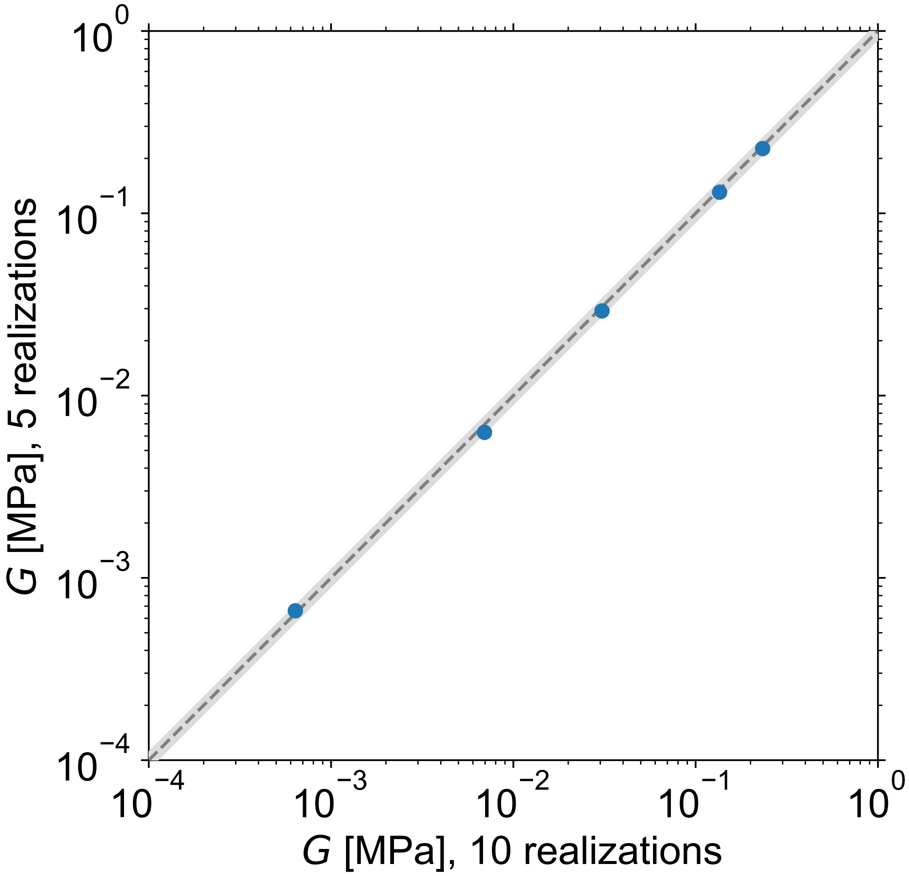

Figure S2: Effect of the number of MC realizations on the equilibrium shear modulus G predicted using the Force Balance procedure. Each MC realization has 10^4 chains. The grey band indicates the $10\,\%$ deviation from the perfect agreement (dashed diagonal line). The input parameters for the systems shown are listed in Table S1.

Table S1: Input parameters for the MC generator for the structures used in Figs. S1 and S2, arranged from the highest to the lowest modulus G. f is the functionality of the cross-links, r the stoichiometric imbalance, w_{sol} the fraction of soluble material, b_2 is the molar fraction of active groups of precursor bifunctional chains in the reactant precursor chain mixture, and N_{B_2} and N_{B_1} denote the number of beads per bifunctional and monofunctional chain, respectively.

System	f	r	w_{sol}	N_{B_2}	N_{B_1}	b_2
1	4	1	0	77	0	1
2	4	1.71	0.012	77	0	1
3	4	1	0	221	227	0.66
4	4	1.44	0.278	221	227	0.68
5	4	0.55	0.638	51	0	1
